# Human blood plasma factors affect the adhesion kinetics of *Staphylococcus aureus* to central venous catheters

**DOI:** 10.1038/s41598-020-77168-x

**Published:** 2020-12-02

**Authors:** Gubesh Gunaratnam, Christian Spengler, Simone Trautmann, Philipp Jung, Johannes Mischo, Ben Wieland, Carlos Metz, Sören L. Becker, Matthias Hannig, Karin Jacobs, Markus Bischoff

**Affiliations:** 1 Institute for Medical Microbiology and Hygiene, Saarland University, Homburg, Germany; 2 Experimental Physics, Saarland University, Saarbrucken, Germany; 3 Clinic of Operative Dentistry and Periodontology, Saarland University, Homburg, Germany; 4 Department of Internal Medicine V, Pneumology and Intensive Care Medicine, Saarland University, Homburg, Germany; 5 Max Planck School Matter to Life, Heidelberg, Germany

**Keywords:** Microbiology, Pathogenesis, Materials science

## Abstract

*Staphylococcus aureus* is a common cause of catheter-related blood stream infections (CRBSI). The bacterium has the ability to form multilayered biofilms on implanted material, which usually requires the removal of the implanted medical device. A first major step of this biofilm formation is the initial adhesion of the bacterium to the artificial surface. Here, we used single-cell force spectroscopy (SCFS) to study the initial adhesion of *S. aureus* to central venous catheters (CVCs). SCFS performed with *S. aureus* on the surfaces of naïve CVCs produced comparable maximum adhesion forces on three types of CVCs in the low nN range (~ 2–7 nN). These values were drastically reduced, when CVC surfaces were preincubated with human blood plasma or human serum albumin, and similar reductions were observed when *S. aureus* cells were probed with freshly explanted CVCs withdrawn from patients without CRBSI. These findings indicate that the initial adhesion capacity of *S. aureus* to CVC tubing is markedly reduced, once the CVC is inserted into the vein, and that the risk of contamination of the CVC tubing by *S. aureus* during the insertion process might be reduced by a preconditioning of the CVC surface with blood plasma or serum albumin.

## Introduction

The gram-positive bacterium *S. aureus* is a common cause of medical device-related infections^1^. The bacterium has the ability to adhere to different kinds of artificial surfaces and to form multilayered biofilms, which are frequently not susceptible to antibiotic therapy^2^. A commonly used endovascular medical device is the central venous catheter (CVC), which is essential in everyday medical practice, especially in treating patients in intensive care units^3^. However, usage of CVCs is also associated with a substantial risk of bloodstream infections (BSIs), and *S. aureus* is currently the major etiological agent of catheter-related bloodstream infections (CRBSIs)^4^. Silicone and polyurethane (PU) are the most commonly used materials for CVC production^5^, with PU displaying a superior permanence over silicone, however, at the cost of increased bacterial colonization rates^5^. A number of studies were conducted in the 1980s and 1990s to elucidate the host and pathogen-related factors responsible for the adhesion of *S. aureus* to CVCs^6–11^. These studies identified cell wall-anchored/associated proteins as important adhesins of *S. aureus* to naïve catheters^9,11,12^. Studies carried out with *Staphylococcus epidermidis* demonstrated that adhesion of the bacterium to the implant material is also driven by surface hydrophobicity and -topology^13–15^, and this probably also holds true for *S. aureus*^16^. Other studies demonstrated that host factors deposited on catheters significantly affect the binding capacity of *S. aureus* to the polymer surfaces^7–10,12,16^. When inserted into the vasculature, CVC surfaces are rapidly (within seconds) covered by plasma proteins, which in turn alter the binding capacity of the bacterium to the implanted medical device^17^. However, there is a certain discrepancy in the literature about the impact of plasma surface coating on the adhesion behavior of *S. aureus* to PU/CVCs. While some studies reported a positive effect of plasma on the adhesion of *S. aureus* to CVCs^9,18^, others observed no^18^ or even a negative effect of plasma or serum on the ability of this bacterium to adhere to PU-based catheters^12,16^. However, based on *S. aureus* adhesion studies performed with untreated and explanted CVCs, it can be assumed that host factors deposited on the CVC upon implantation promote rather than decrease binding of *S. aureus* to the inserted intravascular device^7,8^.

In order to colonize the CVC and to form a biofilm, the bacterium first needs to get into contact with the medical device. This is likely to happen by different means. The bacterium might get into contact with the CVC while the catheter is inserted through the skin. Under these conditions, the bacterial cell residing on the skin is most likely getting into contact with the naïve surface of the CVC via physicochemical and capillary forces^19,20^. It is assumed that most CRBSI originating from short-term CVCs (in place < 10 days) are of cutaneous origin, and gain access extraluminally^3^. A second common route by which *S. aureus* may get into contact with the CVC is contamination of the fluid administered through the device. Under these conditions, native *S. aureus* cells are likely to get into contact with the luminal area of the CVC that is either undecorated (i.e. the rear tubing of the CVC) or decorated by plasma factors (i.e. the front tubing and the tip of the CVC). A third way of CVC colonization by *S. aureus* originates from remote sources of local infection via the bloodstream (hematogenous spread). Under these conditions, *S. aureus* cells that are decorated by plasma factors are likely to get into contact with a CVC surface that is covered with plasma factors as well. Only little information is available yet on how these colonization routes affect the adhesion kinetics between *S. aureus* and the implanted medical device. Similarly, the adhesion forces between the bacterial cell and this type of implant material, and particularly the impact of blood plasma on the initial adhesion of *S. aureus* to CVC surfaces, have to our knowledge not been determined yet. In order to fill these gaps, we applied here a single-cell force spectroscopy (SCFS) approach^21^ to determine the maximum adhesion forces and rupture lengths of viable, native and plasma-coated *S. aureus* cells to naïve and plasma coated PU-based CVCs.

## Results and discussion

### Surface characteristics of PU-based CVC tubing

Our earlier work demonstrated that the adhesion of staphylococci to abiotic surfaces is dominated by hydrophobic/hydrophilic interactions between bacterial cell wall components and the artificial surface^22,23^, and that *S. aureus* displays stronger adhesion forces on hydrophobic than on hydrophilic surfaces^24^. Another important factor that affects the binding capacity of *S. aureus* to an abiotic surface is surface topology^25^. We and others have shown that the adhesion of *S. aureus* to hydrophobized silicon wafers and titanium decreases significantly when the roughness of the surface is increased in the low nanometer (nm) range (≤ 20 nm)^26,27^, while variations in surface topography in the higher nm to micrometer (µm) range (≥ 350 nm) were reported to enhance the bacterial adhesion to a substratum^28,29^.

**Figure 1:**
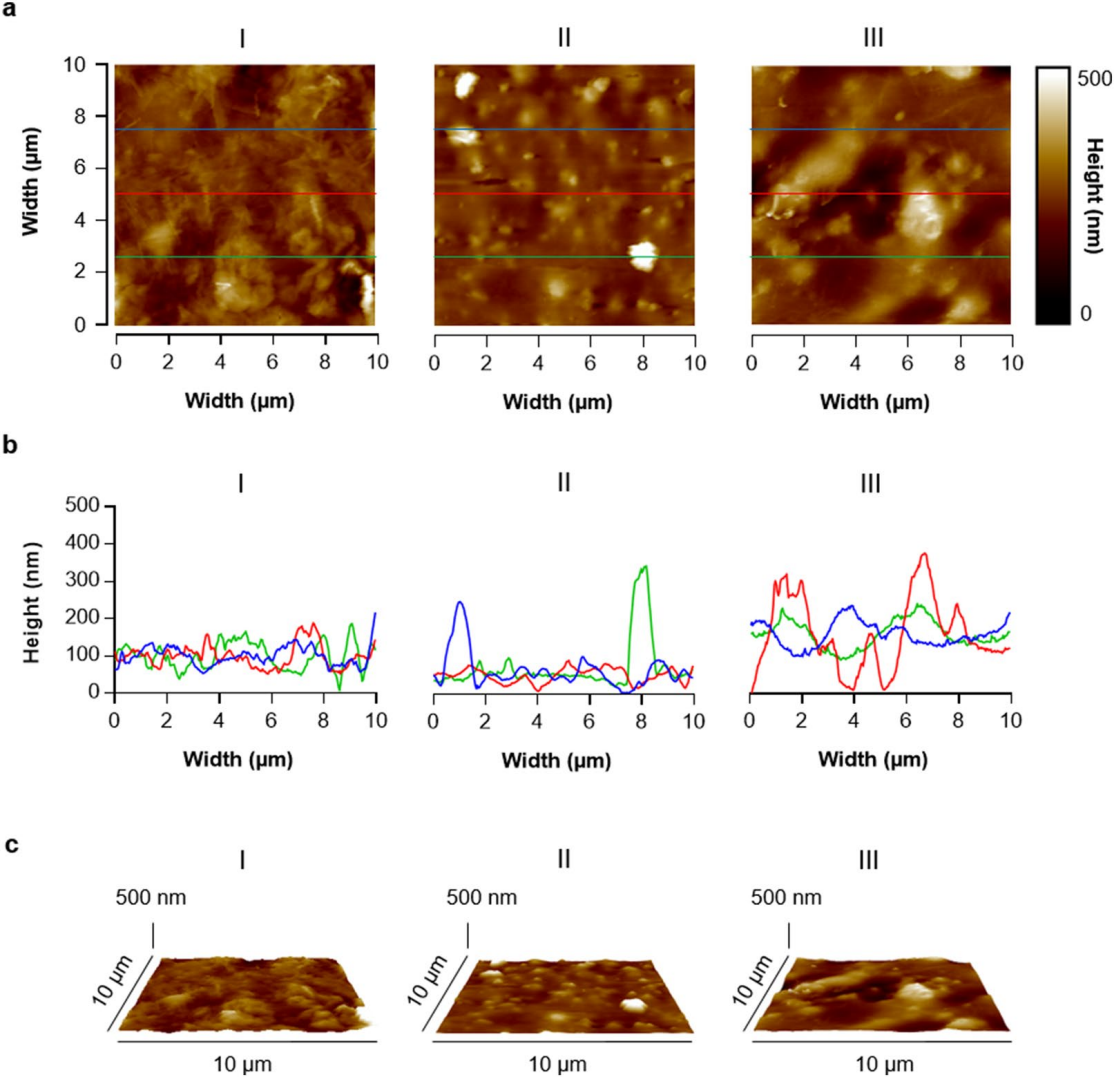
AFM height images of the surfaces of CVC types I to III. 10 × 10 µm areas of the CVC tubes were scanned by AFM as outlined in “Materials and methods” section. (**a**) Representative 2D AFM height images of the CVC types I to III. (**b**) Overlay of height profiles along the lines indicated in (**a**). (**c**) Representative 3D AFM height images of the same regions as displayed in (**a**).

PU-based CVCs are currently the predominant catheter types used for central lines^5^, however, only little is known about the roughness and other surface characteristics of merchandised PU-based CVCs, which may additionally differ substantially between manufacturers. To further elucidate these characteristics, we started our study with the determination of the surface topography and hydrophobicity/hydrophilicity of three CVC types obtained from different manufacturers that are commonly used at the Saarland University Medical Hospital (Fig. 1 and Table 1). All three CVC types displayed advancing water contact angles 90° < Ɵ < 130° (Table 1), indicating that these surfaces are hydrophobic. Similarly, all three CVC tube surfaces exhibited a rather comparable overall roughness, with root mean square (RMS) roughness values between 51 and 58 nm (Table 1). However, the 10 µm × 10 µm height profiles recorded by AFM indicated that the three CVC types display differences in their surface topologies (Fig. 1). CVC surfaces of type III displayed noisy surface topologies with multiple small peaks and numerous bumps and valleys with height differences up to 500 nm. Type II CVC surfaces exhibited flatter surface topologies with a number of smaller but high bumps, and type I CVC surfaces displayed again more bumpy surface topologies, however, with the smallest variations between bumps and valleys (Fig. 1). When the skewness, the degree of asymmetry of a surface height distribution, was calculated from the AFM images, CVC surfaces of type II featured the highest skewness values, while CVC surfaces of types I and III displayed lower values that were in a comparable range (Table 1), in line with earlier findings suggesting CVC type II to exhibit a more irregular surface topology than CVC types I and III^15^.

**Table 1:** Surface characteristics of the tubing of CVC types I–III.

	CVC type		
	I	II	III
Advancing water contact angle Ɵ (°)	119 ± 6	109 ± 1	109 ± 1
Root-mean-square roughness (nm)	57.5 ± 6.9	50.8 ± 7.1	58.3 ± 18.3
Skewness	0.64 ± 0.77	1.69 ± 0.67	0.44.3 ± 0.1

Data are represented as mean ± SD.

### *S. aureus* displays comparable adhesion forces on different types of PU-based CVC surfaces

**Figure 2:**
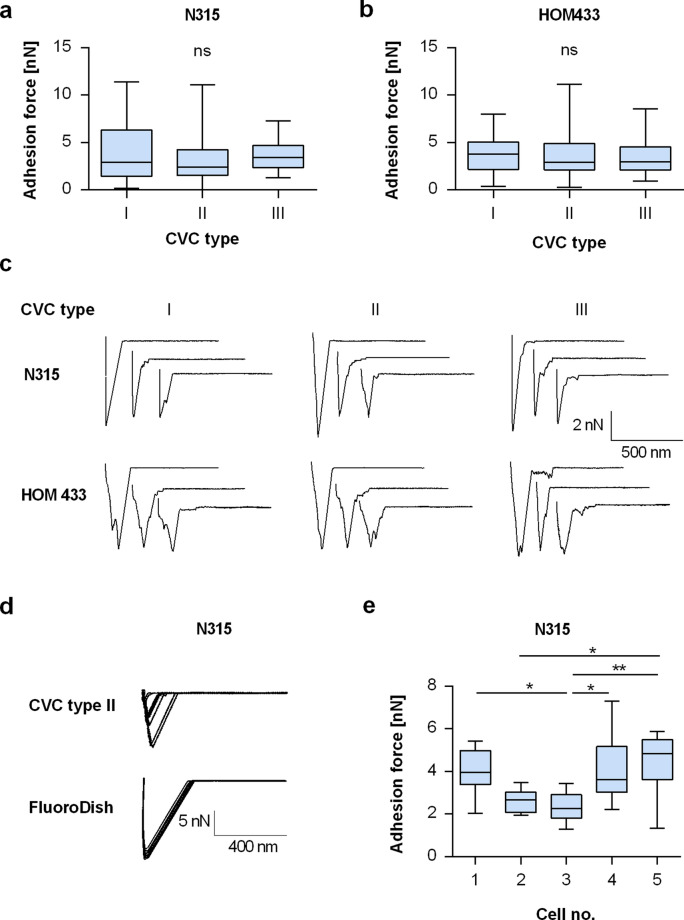
Adhesion kinetics of *S. aureus* on naïve CVC types I to III. Individual exponential growth phase cells of *S. aureus* were immobilized on cantilevers and used for SCSF on naïve surfaces of CVC types I to III. (**a**,**b**) Maximum adhesion forces extracted from the retraction curves recorded by SCFS with cells of strains N315 (a) and HOM433 (b) on the naïve surfaces of CVC types I to III. Data are presented as box and whisker plots (min-to-max) of 50 values obtained with 5 individual cells per strain. ns, not significant (Kruskal–Wallis test followed by Dunn’s post hoc test). (**c**) Representative retraction curves collected from cells that were probed with naïve CVC surfaces from manufacturers I to III. (**d**) Overlays of 10 retraction curves obtained with a single N315 cell on glass and the surface of CVC type II, respectively. (**e**) Maximum adhesion forces of cells of strain N315 on the surface of CVC type III. Data are presented as box and whisker plots showing the interquartile range (25–75%; box), median (horizontal line), and whiskers (bars; min/max) of 10 values obtained per individual cell. *P < 0.05; **P < 0.01 (Kruskal–Wallis test followed by Dunn’s post hoc test).

Although the quantitative and qualitative adhesion of staphylococci to CVCs has been addressed by many studies, the adhesion forces between *S. aureus* and this type of implant material have to our knowledge not been determined yet. Thus, we next studied the adhesion behavior of viable *S. aureus* cells to the CVC surfaces by SCFS, which allows measuring the interaction forces between a single bacterial cell and its substratum with pN force resolution^21^. In line with our surface characterization experiments (Fig. 1 and Table 1), we detected rather comparable maximum adhesion forces (F_adh_) for *S. aureus* strain N315 on all three types of CVCs (Fig. 2a), which averaged between 3.1 ± 2.3 nN and 4.0 ± 2.9 nN (mean and SD). N315 cells tended to exhibit lower adhesion forces on the CVC surface of type II, however, this effect was not statistically significant. A similar trend was observed for the rupture lengths obtained with N315 on the CVC surface of type II, which were about half as long as the ones observed with this strain on the CVC surface of type III (Table S1).

Since an earlier study noticed larger strain to strain variations in the capacity of *S. aureus* to adhere to CVC surfaces^16^, we conducted a second series of SCFS measurements with the CRBSI-related clinical isolate HOM433 to exclude that the findings made with N315 might be restricted to this strain. When cells of the clinical isolate were probed on the three naïve CVC surfaces, very similar F_adh_ values and rupture lengths were observed on the respective CVC types (Fig. 2b and Table S1). The vast majority of the recorded retraction curves obtained with the two isolates displayed a clear main detachment peak with only a few discrete steps (Fig. 2c), suggesting the involvement of numerous bacterial adhesins and a rather abrupt detachment of the bacterial cell surface components from the CVC surface^23,24^. The maximum adhesion forces recorded for *S. aureus* N315 on PU-based CVC surfaces in this study were in a similar range to those observed for this bacterium on silicone rubber^30^, and about a factor 10 higher than those found on titanium (J. Mischo, unpublished results), a commonly used material for orthopedic and dental implants^31^. This suggests that *S. aureus* exhibits a comparably strong binding capacity to naïve PU-based CVC surfaces.

In contrast to a previous report on the adhesion of *S. aureus* to hydrophobized Si wafers with a very smooth surface topography (RMS of 0.12 nm), where the force–distance measurements with an individual bacterial cell provided highly superimposable retraction curves^23^, we observed comparably large variations in the maximum adhesion forces recorded for some of the individual cells (Table S1). One reason for this might be the surface roughness of the CVCs tubes tested. In biophysical studies, SCFS is commonly carried out on highly defined and very smooth surfaces lacking larger bumps, while our test surfaces displayed numerous smaller and larger humps (Fig. 1). To access the impact of surface roughness on the adhesion force variation seen between repeated measurements with the identical bacterial probe, we additionally determined the maximum adhesion forces for N315 cells on FluoroDish, a hydrophobic glass surface^32^ displaying a low surface roughness (RMS = 1.1 ± 0.2 nm) and lacking any major humps. On this type of glass surface, N315 produced retraction curves and adhesion forces that were highly similar (Fig. 2d), suggesting that surface roughness might indeed be a factor contributing to the large variation in adhesion forces seen for individual *S. aureus* cells on the CVC surfaces in repeated measurements. Another explanation for this phenomenon might be chemical and biomechanical variations in surface composition. PU-based materials are formed in block copolymers creating a two phase microstructure composed of 10–100 nm microdomains with different physicochemical properties^33^. It has been shown that these microdomains display differences in the adsorption of plasma proteins and cell attachment^34^.

Similar to the variations in adhesion forces seen for repeated measurements with the same bacterial probe, we also noticed in part significant variations in the mean adhesion forces between individual cells (Fig. 2e and Table S1). This observation is rather common for SCFS studies with staphylococci^22,24,26,31,35^ and probably due to cell-to-cell variations that occur even between individual cells sampled from the same cell culture and at the same time point.

### Precoating of the CVC surface with human blood plasma decreased the adhesion force of *S. aureus* to CVC surfaces

**Figure 3:**
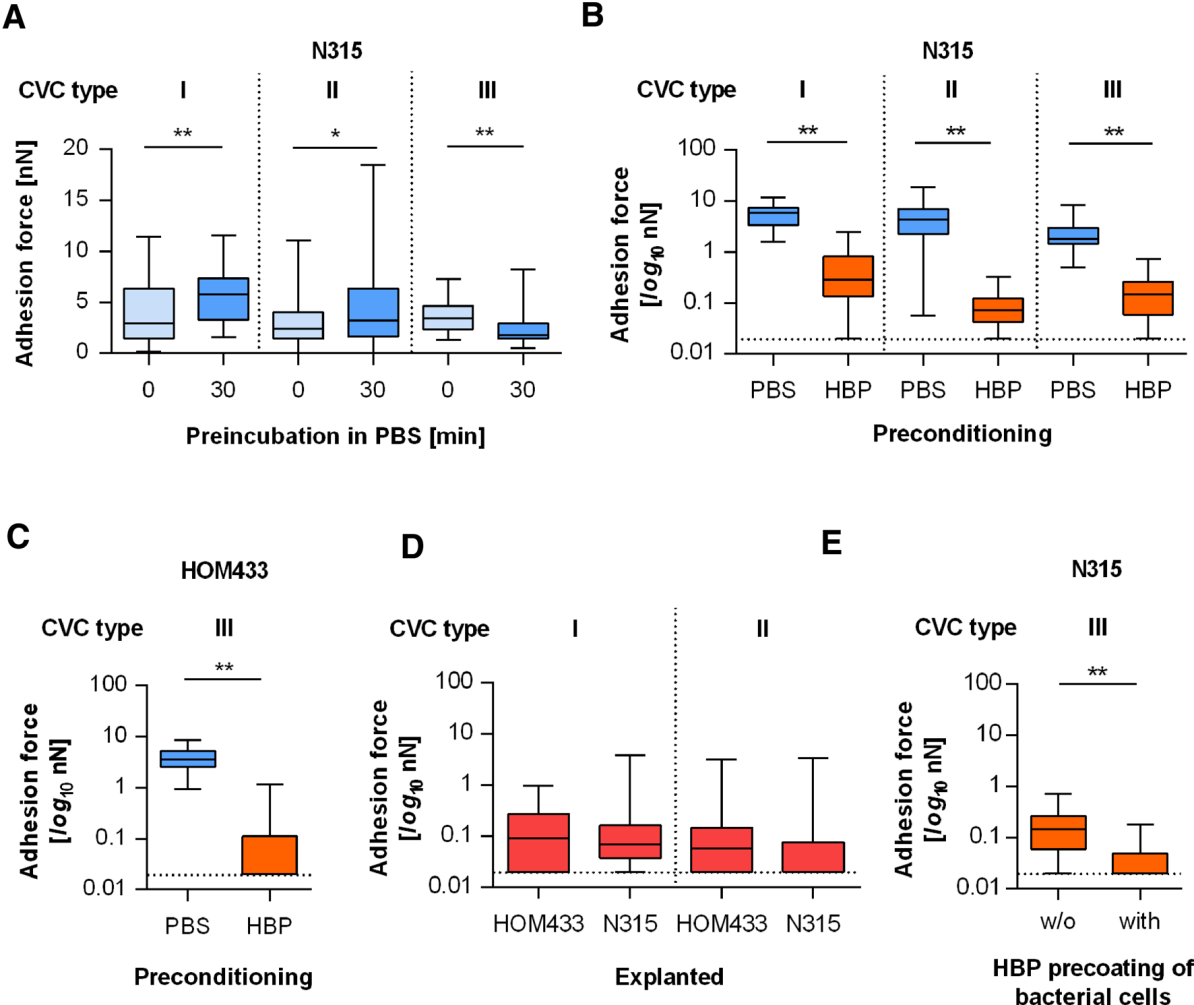
Adhesion kinetics of *S. aureus* on precoated CVC surfaces. Individual exponential growth phase cells of *S. aureus* strains N315 and HOM433 were used for SCSF on naïve and pretreated CVC surfaces. (**A**) Impact of a PBS preincubation (30 min) on the maximum adhesion forces of N315 cells on the surfaces of CVC types I to III. (**B**) Impact of a HBP preconditioning (30 min) on the maximum adhesion forces of N315 on the surfaces of CVC types I–III. (**C**) Impact of a HBP pretreatment (30 min) on the maximum adhesion forces of HOM433 on the surface of CVC type III. (**D**) Maximum adhesion forces of untreated N315 or HOM433 cells on the surface of freshly explanted CVCs of types I and II obtained from patients without CRBSI. (**E**) Impact of a HBP preconditioning of N315 cells on the maximum adhesion force of the bacterium formed on HBP-precoated surfaces of CVC type III. Data are presented as box and whisker plots (min-to-max) of 40–60 values obtained with 4–6 individual cells per condition. The detection limit of our system is indicated by horizontal dashed lines. *P < 0.05; **P < 0.01 (Mann Whitney *U* test).

When inserted into the body, blood-exposed implanted materials are decorated within seconds by blood plasma factors^36^, but it is a matter of debate whether this positively or negatively affects the adhesion capacity of *S. aureus* to the implanted medical device^9,16,18,37^. Additionally, the impact of blood plasma on the initial adhesion of *S. aureus* to CVC surfaces has not been determined yet. In order to address this issue, we performed SCFS with *S. aureus* N315 on CVC fragments that were preincubated for 30 min with human blood plasma (HBP). Since PU-based materials are known to alter their surface properties in aqueous solutions^34^, we first determined the maximum adhesion forces for N315 on the surfaces of CVC tubes that were precultured for 30 min in PBS. Although no clear signs of swelling were observed for this comparably short time period, we noticed significant differences in the F_adh_ values on PBS preincubated CVC surfaces when compared to naïve CVC surfaces (Fig. 3A). This suggests that the contact with aqueous solutions for 30 min already alters the surface properties of PU-based CVCs in a way that promotes (CVC types I and II) or decreases (CVC type III) *S. aureus* adhesion.

When force–distance curves of strain N315 were recorded on catheter surfaces that were preincubated for 30 min with HBP, significant decreases in F_adh_ values were observed for all three CVC types (Fig. 3B), which dropped by ≥ tenfold when compared to the F_adh_ values obtained on the PBS preincubated CVC surfaces of the same type. To substantiate this observation, we carried out a second set of experiments with strain HOM433 on surfaces of CVC type III (Fig. 3C), which yielded a very similar result. These findings strongly suggest that the adhesion capacity of *S. aureus* to this type of medical device is drastically reduced, once the CVC is inserted into the vein, and support earlier work reporting a negative effect of plasma or serum on the adhesion of *S. aureus* to polymer materials^12,16^.

One factor that might explain the lower F_adh_ values observed for *S. aureus* on HBP-precoated CVC surfaces is surface hydrophobicity, as *S. aureus* displays a reduced adhesion capacity to hydrophilic surfaces^24^. Thus, we next determined the impact of a HBP preconditioning on the wettability of the CVC tubing. HBP-pretreated surfaces of CVC types I, II and III displayed advancing water contact angles of 55 ± 4°, 39 ± 4° and 51 ± 5°, respectively. These findings suggest that a HBP preconditioning renders the surface of the CVC tubing from hydrophobic to hydrophilic, and that this change in wettability might indeed contribute to the reduced maximum adhesion forces observed for *S. aureus* on the HBP-precoated CVC tubing. Specific receptor–ligand interactions between bacterial cell wall proteins and blood plasma factors deposited on the CVC tubing, on the other hand, seem to be of minor importance, albeit of the fact that *S. aureus* expresses a number cell wall-anchored or -associated adhesins capable of binding to blood plasma factors such as fibrinogen (Fg), fibronectin, immunoglobulin G, vitronectin, and von Willebrand factor (vWF), respectively^38^, which all were reported to adsorb onto the surfaces of implant materials upon contact of the foreign material with blood^39,40^.

### *S. aureus* displayed low adhesion forces on explanted CVC tubings

Flow conditions and dwelling times are both known to affect the composition of the plasma factor coating on implanted materials^36^ and to induce conformational changes of the adsorbed proteins^34^. To see if and how our findings received with the in vitro HBP preincubated CVC surfaces can be matched with the in vivo situation, we next tested the adhesion forces of strains N315 and HOM433 on freshly explanted CVC tubing withdrawn from patients that did not suffer from CRBSI. When force–distance curves were recorded on this type of material, we observed maximum adhesion forces (Fig. 3D) that were very similar to the F_adh_ values determined with N315 and HOM433 on the in vitro HBP preincubated CVC surfaces (Fig. 3B,C), suggesting that our findings received with the in vitro test system can be correlated with the in vivo situation.

### Coating of the *S. aureus* cell wall with HBP factors further reduced the adhesion force of the bacterial cell to the HBP-precoated CVC surface

A third route of CVC colonization by *S. aureus* originates from hematogenous spreading of the bacterium from other infection foci^41^. Under these conditions, *S. aureus* cells that are decorated by plasma factors and/or attached to blood cells get into contact with a CVC surface that is covered with plasma factors and blood cells as well. In order to mimic this scenario, we preincubated N315 cells for 30 min with HBP before we recorded the force–distance curves with such cells on HBP-precoated CVC fragments of type III (Fig. 3E). The preconditioning of the bacterial cell with HBP further reduced the maximum adhesion force between N315 and the HBP-precoated CVC surface, when compared to the adhesion forces observed for uncoated N315 cells on HBP preincubated CVC surfaces. These findings suggest that blood borne *S. aureus* cells exhibit a rather lower adhesion capacity to CVC surfaces inserted into the vasculature, at least under static conditions. However, our experimental setup lacks at least two major factors relevant for the adhesion of *S. aureus* to implant material that is inserted into the vasculature, which are the presence of blood cells, e.g. platelets bound to the catheter surface that might be exploited by the bacterium for adhesion, and shear flow. *S. aureus* cells are reported to adhere to platelets with high efficiency^42^, and platelets adhere to implanted material inserted into the bloodstream^43^. Although it is unknown yet to which extent *S. aureus* adheres to blood-exposed implant material via platelets, it can be assumed that platelets contribute to the adhesion and biofilm formation of *S. aureus* to/on CVCs, given the interplay between *S. aureus* and this type of blood cells^42^. Another factor that is likely to be important for the binding capacity of *S. aureus* to implant material that is inserted into the vasculature is shear flow. Most regions of the CVC tubing that are inserted into larger veins are exposed to high shear stress^44^, and we and others have shown that binding of *S. aureus* to activated endothelium under high shear flow is mediated predominantly via elongated vWF fibers^45,46^. vWF unfolding is only observed when vWF is tethered to a surface and exposed to high shear stress^47^. vWF is also known to adsorb on blood-exposed catheters^40^. Although vWF seems to be of minor importance for platelet adhesion on blood-exposed implant material^48^, its elongated form is likely to contribute to the adhesion capacity of *S. aureus* to the blood-exposed implant material under high shear flow. To access such shear flow-induced phenomena, the actual flow profile must be precisely determined (e.g. in a microfluidic setup with particle imaging velocimetry), which is beyond the scope of the present study.

### The adhesion force of *S. aureus* to CVC surfaces is reduced by a preincubation of the CVC tubing with serum albumin or Fg

**Figure 4:**
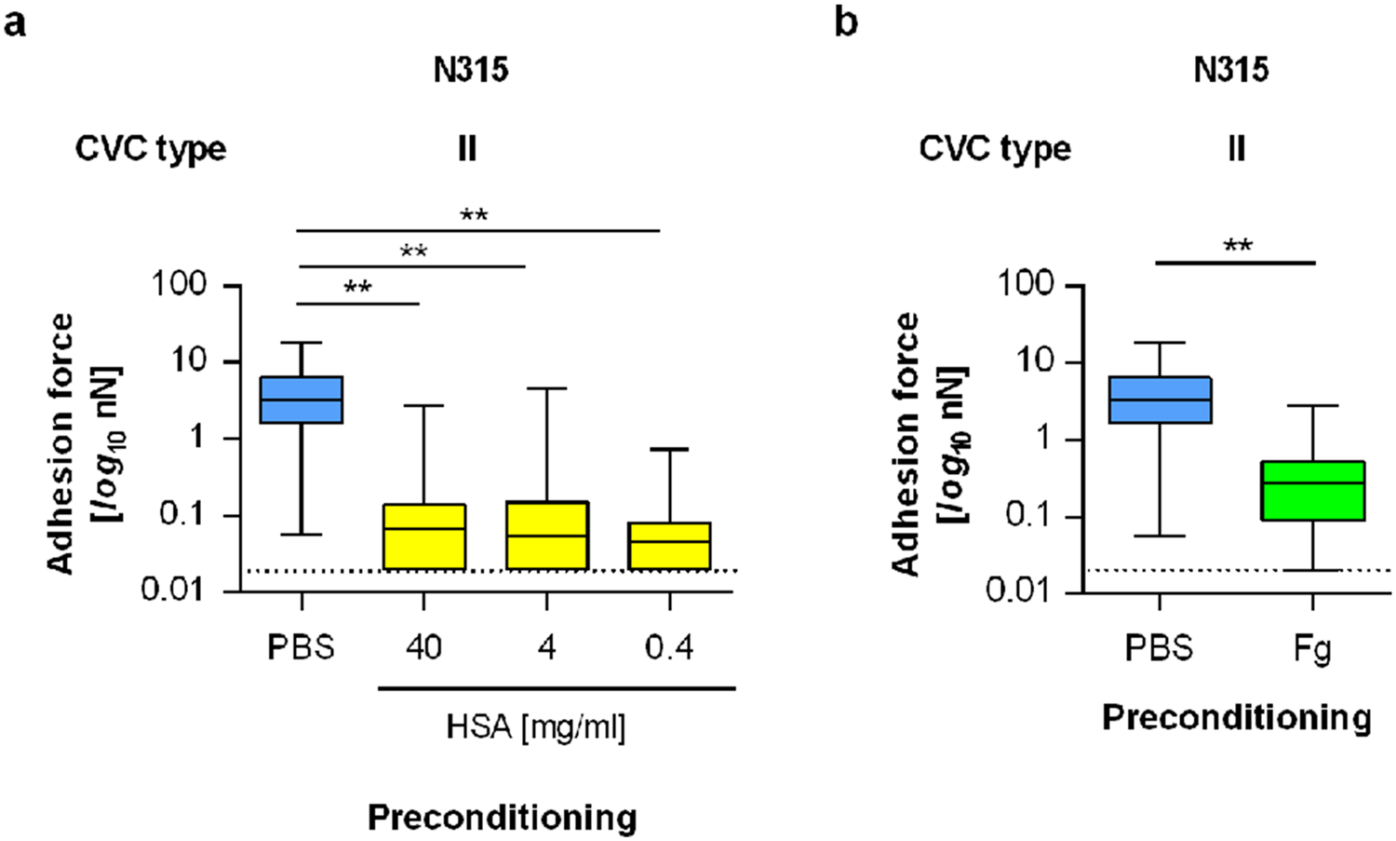
Impact of HBP-factors on the adhesion of *S. aureus* to CVC tubing. Individual exponential growth phase cells of *S. aureus* strain N315 were used for SCSF on naïve and pretreated tubing of CVC type II. (**a**) Impact of human serum albumin (HSA) on the maximum adhesion forces of untreated N315 cells formed on HSA (0.4, 4, and 40 mg/ml for 30 min) pretreated CVC surfaces. (**b**) Impact of human fibrinogen (Fg) on the maximum adhesion forces of untreated N315 cells formed on Fg (0.4 mg/ml for 30 min) precoated CVC surfaces. Data are presented as box and whisker plots (min-to-max) of 60 values obtained with 6 individual cells per condition. The detection limit of our system is indicated by dashed lines. **P < 0.01 (Kruskal–Wallis test followed by Dunn’s post hoc test [a] or Mann Whitney *U* test [b]).

Serum albumin and Fg are the main proteinaceous plasma factors that are adsorbed on blood-exposed polymer surfaces^49^, and the preincubation of a CVC with serum albumin has been shown to reduce the adhesion of *S. aureus* to the CVC surface^8^. In line with the latter publication, we also found a strong decrease in the maximum adhesion forces between *S. aureus* N315 and the catheter surface of type II when the catheter fragment was preincubated with human serum albumin (HSA; Fig. 4a). The adhesion forces of N315 on the CVC surface dropped by a factor of about 35 when the catheter fragments were preincubated with 40 mg/ml of HSA, a common albumin concentration found in human blood^50^. Notably, similar reductions in the mean F_adh_ values were observed even when HSA concentrations as low as 0.4 mg/ml were used for preincubation (Fig. 4a), suggesting that much lower concentrations of this blood plasma protein than the ones usually found in human blood are sufficient to markedly suppress the initial adhesion capacity of *S. aureus* N315 to PU-based CVC surfaces.

A smaller, but still highly significant reduction in mean F_adh_ values was also observed when N315 cells were probed with CVC fragments of type II that were preincubated with physiological concentrations of human Fg (Fig. 4b). The latter observation is surprising since earlier work reported positive effects of Fg on the adhesion capacity of *S. aureus* to implanted materials in quantitative adhesion assays^7,51^. Given these differences in the impact of Fg on the qualitative and quantitative adhesion of *S. aureus* to implanted material in vitro, we hypothesize that a decoration of the CVC surface with physiological concentrations of Fg reduce the interaction force between *S. aureus* and the implanted material, while the quantitative adhesion capacity of *S. aureus* to this type of implanted material is promoted by the presence of this host factor on the CVC surface. This scenario might also hold true for human blood plasma factors adsorbed on the CVC surface, which were found here to strongly decrease the adhesion forces between *S. aureus* and PU-based CVCs, while others observed enhanced numbers of *S. aureus* cells on different types of catheter surfaces that were withdrawn from patients^8^. However, in the latter study, the quantitative adhesion of *S. aureus* to explanted catheter fragments was compared to catheter fragments that were precoated with serum albumin, leaving the question open to which extent *S. aureus* would have adhered to the respective naïve catheter fragments.

### Concluding remarks

To our knowledge, this is the first time that SCFS with *S. aureus* was done on authentic, commercially available CVC material to determine the adhesion forces between a commonly used implanted medical device and this major nosocomial pathogen. We showed that *S. aureus* binds with high affinity to PU-based CVC tubing and that this adhesion can be markedly reduced by a pretreatment of the CVC tubing with blood plasma or serum albumin. The preconditioning of the CVC surface with serum albumin, a procedure suggested to reduce the risk of thrombus formation on blood exposed medical devices^52^, might also be a valid tool to decrease the risk of contamination of the CVC tubing with *S. aureus*, particularly during the insertion process. The reduced adherence strength of *S. aureus* cells to blood plasma precoated PU-based CVC tubing is probably due to a decrease in surface hydrophobicity of the implant material evoked by the adsorbed plasma factors. This assumption is in line with earlier findings made with *S. aureus* and different types of venous catheters demonstrating that chemical modifications which rendered the catheters more hydrophilic also decreased the quantitative adhesion of the bacterium to the modified surfaces^18,53,54^, and supports the idea that increasing the hydrophilicity of PU-based CVC tubing might be a valuable tool to lower the risk of bacterial colonization of this implanted medical device^6^. Our data suggest furthermore that a chemical modification of the tubing surface might not be needed to achieve this goal.

## Materials and methods

### Bacteria and bacterial probes

*S. aureus* strain N315 was obtained from the Network on Antimicrobial Resistance in *S. aureus* (NARSA) strain collection (Catalog Number: NRS70). The clinical *S. aureus* isolate HOM433 was collected from a CVC that was removed from a patient suffering from a catheter-related *S. aureus* blood stream infection at the Saarland University Hospital, Homburg, Germany. The bacteria were grown on Tryptic Soy Agar Plates with 5% sheep blood (Becton Dickinson [BD], Heidelberg, Germany), and subsequently multiplied in Tryptic Soy Broth (TSB, BD) in Erlenmeyer flasks using a culture to flask volume of 1:10 for 16 h at 37 °C and 150 rpm. On the next day, the bacterial cell number was set to an optical density at 600 nm of 0.05 in TSB and bacterial cells were grown for 2.5 h at 225 rpm to the exponential growth phase. After harvesting, bacterial cells were centrifuged for 1 min at 12,000 rpm and were washed twice with phosphate buffered saline (PBS, pH 7.4) to remove debris and extracellular material. Bacteria were then prepared for AFM and diluted 1:50 in PBS, where bacterial aggregates were separated with an ultrasonic probe (B. Braun, Melsungen, Germany) for 15 s at 50 W. Next, a single bacterium was attached to a polydopamine-coated tipless AFM cantilever (MLCT-0 with a nominal spring constant of 0.03 N m^−1^ from Bruker-Nano, Santa Barbara, USA) via a micromanipulator (Narishige Group, Tokyo, Japan) as described in^21^. The functionalization of the cantilever was controlled optically with an inverted microscope and by force–distance measurement on a glass-bottomed petri dish (FluoroDish FD35-100; World Precision Instruments, Friedberg, Germany). The viability of the bacterial cell at the end of the force/distance measurements was confirmed by live/dead staining using the LIVE/DEAD *Bac*Light Bacterial Viability Kit (Thermo Fisher Scientific, Dreieich, Germany).

### Human samples

Human blood was obtained from healthy volunteers older than 18 years of age. Informed written consent was obtained from all participants. Explanted CVCs were obtained from the Department of Internal Medicine V, Pneumology and Intensive Care Medicine, Saarland University, Homburg, Germany. The study design was approved by the Ethics Committee of the Medical Association of Saarland (code numbers 95/18 and 39/20) before recruitment and study initiation. The study was performed according to the guidelines of the Declaration of Helsinki. The acquisition procedures of blood and explanted CVC samples, respectively, were checked and approved by the local ethics committee.

### Substrate preparation

CVC tubing samples were prepared from the Arrow CVC set DE-14703-S (Teleflex Medical GmbH, Fellbach [type I]), B. Braun Certofix Trio S 720 (B. Braun, Melsungen, Germany [type II]), and Vygon Seldipur Safe (Vygon, Aachen, Germany [type III]). Catheter packages were opened and CVCs handled in a level 2 biosafety cabinet to reduce contaminant deposition on the catheter surface. The CVC tubing was cut with a sharp scalpel into ~ 0.5 cm fragments, and single fragments were attached to a flat glass-bottom petri dish with a water-resistant glue (Pattex, Düsseldorf, Germany), which was allowed to harden for 30 min under the BSC to strengthen the fixation between catheter and glass surface. For the fixation of explanted CVC fragments, sample fixation was done the same way, but the glass petri dish was additionally placed in a humidity chamber to prevent the biological coating on the CVC from drying. HBP was derived from freshly withdrawn blood collected in S-Monovette lithium-heparin blood collection tubes (Sarstedt, Nümbrecht, Germany) that was centrifuged at 5000 rpm for 2 min. HBP was transferred to a fresh reaction tube, centrifuged one more time under the same conditions to remove any cell material, and then stored in a fresh reaction tube at − 80 °C until usage.

### Surface characterization of the CVC tubing

For mapping of the height topography of the CVC tubing, fragments of the three CVC types were scanned in PBS in Peak Force Tapping intermittent contact mode with an atomic force microscope (Bioscope Catalyst; Bruker Nano). For measurements in PBS, silicon nitride tips (SNL-10 with nominal spring constant of 0.35 N/m from Bruker-Nano) were used. Typically, an area of 10 µm × 10 µm (256 × 256 pixels) was scanned with a scan rate set to 0.1 Hz. The resulting images were flattened (second order) and plane fitted (first order), and used to determine roughness parameters with the Nanoscope Analysis software 1.9 (Bruker Nano). Water contact angle determinations on CVC surfaces were done as follows: From each CVC type, 2 cm long pieces were cut off and mounted on a straight needle. Pieces were immersed into ultrapure water at a constant velocity of 1 mm/s using an automated setup. At the same time, the water surface around the CVC pieces was recorded with a camera from the direction perpendicular to the line of movement at frame rates of 10 frames per second. Afterwards, the CVC pieces were placed in HBP for 30 min at 37 °C, subsequently rinsed 8-times with water, and the wettability of the precoated surfaces recorded as described above. The water contact angles before and after plasma coating were determined from every frame of the recorded movies using a self-written Matlab script in which the edge of the CVC was fitted with a linear function and the water surface with a third-order polynomial.

### Single-cell force spectroscopy

CVC fragments were attached to flat-bottom glass petri dishes as outlined above, covered with PBS, and measurements were started within five min to mimic the adhesion of single bacterial cells to the naïve surface. The bacterial probe was placed above the center of the catheter, and carefully approached towards the surface. 10 force–distance curves were recorded per bacterial probe on the crest of each CVC fragment with a force trigger set to 300 pN, a lateral distance between two force–distance curves set to 1 µm, the ramp velocity adjusted to 160 nm/s and a ramp size of 800 nm. For the preconditioning of the CVC tubing with HBP (derived from whole blood withdrawn from at least three healthy volunteers), HSA (purchased from Merck Millipore, Darmstadt, Germany), or human Fg (purchased from Sigma-Aldrich, Steinheim, Germany), fixed CVC pieces were covered with a droplet of HBP, HSA (0.4, 4, and 40 mg/ml), or Fg (0.4 mg/ml), respectively, incubated for 30 min at 37 °C, and subsequently washed eight times with PBS to remove loosely attached plasma factors/proteins that might distort the AFM measurements. Precoated CVC fragments were again covered in PBS, and force–distance curves recorded as outlined above. Analysis and depiction of force–distance curves were done with the Nanoscope Analysis software and OriginPro 2019b (OriginLab Corporation, Northampton, MA, USA).

### Statistical analyses

The statistical significance of changes between groups was assessed with Kruskal–Wallis tests followed by Dunn’s post-hoc tests (3 and more groups) or the Mann–Whitney *U* test (two groups) using the GraphPad software package Prism 6.01. *p* values < 0.05 were considered statistically significant.

## Supplementary information

Supplementary information.

## Data Availability

The datasets generated during and/or analyzed during the current study are available from the corresponding author on reasonable request.
