# Publisher Correction: Human blood plasma factors affect the adhesion kinetics of *Staphylococcus aureus* to central venous catheters

**DOI:** 10.1038/s41598-021-89018-5

**Published:** 2021-05-04

**Authors:** Gubesh Gunaratnam, Christian Spengler, Simone Trautmann, Philipp Jung, Johannes Mischo, Ben Wieland, Carlos Metz, Sören L. Becker, Matthias Hannig, Karin Jacobs, Markus Bischoff

**Affiliations:** 1 Institute for Medical Microbiology and Hygiene, Saarland University, Homburg, Germany; 2 Experimental Physics, Saarland University, Saarbrucken, Germany; 3 Clinic of Operative Dentistry and Periodontology, Saarland University, Homburg, Germany; 4 Department of Internal Medicine V, Pneumology and Intensive Care Medicine, Saarland University, Homburg, Germany; 5 Max Planck School Matter To Life, Heidelberg, Germany

Correction to: *Scientific Reports* 10.1038/s41598-020-77168-x, published online 02 December 2020

The original PDF version of this Article contained an error in the spelling of the author Sören L. Becker which was incorrectly given as Becker Sören L.

This has now been corrected in the PDF; the HTML version of the paper was correct from the time of publication.

