# Supplementary material for: Human blood plasma factors affect the adhesion kinetics of Staphylococcus aureus to central venous catheters
Source: Sci Rep. 2020 Dec 2;10:20992. doi: 10.1038/s41598-020-77168-x (PMC7710740; doi:10.1038/s41598-020-77168-x)
Supplement: Supplementary file 1 — Supplementary information. [file 41598_2020_77168_MOESM1_ESM.pdf]

Supplementary Information

Human blood plasma factors affect the adhesion kinetics of *Staphylococcus aureus* to central venous catheters

Gubesh Gunaratnam[1], Christian Spengler[2], Simone Trautmann[3], Philipp Jung[1], Johannes Mischo[2], Ben Wieland[1], Carlos Metz[4], Sören Becker[1], Matthias Hannig[3], Karin Jacobs[2,5], and Markus Bischoff [1*]

[1]Institute of Medical Microbiology and Hygiene, Saarland University, Homburg, Germany

[2]Experimental Physics, Saarland University, Saarbrucken, Germany

[3]Clinic of Operative Dentistry and Periodontology, Saarland University, Homburg, Germany.

[4]Department of Internal Medicine V, Pneumology and Intensive Care Medicine, Saarland University, Homburg, Germany

[5]Max Planck School Matter to Life, Heidelberg, Germany

***Correspondence:**

Dr. Markus Bischoff

markus.bischoff@uks.eu

Table S1: Adhesion forces between *S. aureus* and the naïve tubing of catheter types I-III

	**N315**		**HOM 433**	
**Cell No.**	**F_{adh} (nN)**	**L_{rupt} (nm)**	**F_{adh} (nN)**	**L_{rupt} (nm)**
	**Catheter type I**			
**1**	1.1 ± 0.3	229 ± 57	3.1 ± 1.2	148 ± 37
**2**	6.8 ± 3.5	212 ± 170	2.7 ± 1.8	212 ± 36
**3**	3.9 ± 1.7	150 ± 46	5.3 ± 1.2	152 ± 27
**4**	3.1 ± 1.4	88 ± 23	3.8 ± 1.2	147 ± 24
**5**	4.9 ± 3.1	176 ± 79	4.8 ± 1.8	229 ± 56
**M±SD**	**4.0 ± 2.9**	**171 ± 101**	**3.6 ± 1.6**	**180 ± 53**
	**Catheter type II**			
**1**	1.9 ± 1.2	77 ± 38	3.6 ± 1.9	101 ± 27
**2**	2.3 ± 1.6	106 ± 39	4.6 ± 2.1	185 ± 66
**3**	5.7 ± 3.3	189 ± 62	3.8 ± 3.5	330 ± 191
**4**	3.2 ± 1.1	116 ± 49	2.4 ± 0.5	210 ± 100
**5**	2.5 ± 1.6	141 ± 40	2.8 ± 1.1	229 ± 81
**M±SD**	**3.1 ± 2.3**	**125 ± 59***	**3.5 ± 2.2**	**211 ± 127**
	**Catheter type III**			
**1**	3.9 ± 1.1	157 ± 50	5.8 ± 1.8	229 ± 68
**2**	2.6 ± 0.6	176 ± 42	3.1 ± 1.7	225 ± 76
**3**	2.3 ± 0.7	215 ± 11	2.8 ± 1.0	180 ± 95
**4**	4.0 ± 1.5	425 ± 159	3.9 ± 1.2	1021 ± 156
**5**	4.6 ± 1.4	176 ± 73	1.6 ± 0.5	221 ± 46
**M±SD**	**3.5 ± 1.4**	**231 ± 129**	**3.5 ± 1.9**	**226 ± 79**

Maximum adhesion force (F_{adh}) and rupture length (L_{rupt}) measured between single *S. aureus* N315 or HOM 433 bacteria and the naïve tubing surface of catheter types I to III (10 measurements per cell and catheter type recorded on a 10 µm line on the top of the tubing). *, P<0.05 between CVC type II and CVC types I and III, respectively (Kruskal-Wallis test followed by Dunn's post hoc test)
